# A Rare Case of Chronic Myelogenous Leukemia Presenting as T-Cell Lymphoblastic Crisis

**DOI:** 10.1155/2018/7276128

**Published:** 2018-11-18

**Authors:** Parikshit Padhi, Margarita Topalovski, Radwa El Behery, Eduardo S. Cantu, Ramadevi Medavarapu

**Affiliations:** ^1^Department of Hematology and Medical Oncology, Memorial Medical Center, Las Cruces, NM, USA; ^2^Department of Pathology, Memorial Medical Center, Las Cruces, NM, USA; ^3^Hematopathologist, Integrated Oncology, Phoenix, AZ, USA; ^4^Senior Clinical Laboratory Directory, Cytogenetics, Integrated Oncology, Phoenix, AZ, USA; ^5^Assistant Professor, Hematology and Oncology, University of New Mexico, Memorial Cancer Center, Las Cruces, NM, USA

## Abstract

Chronic Myelogenous Leukemia in blast crisis can manifest as either myeloid (more common) or lymphoid blast crisis. Most lymphoblastic crises are of B-cell lineage. T-cell blast crisis is extremely rare, with only a few reported cases. We present a case of a middle-aged man who was diagnosed with CML on peripheral blood and bone marrow biopsy. Because of a generalized lymphadenopathy noted at the time of diagnosis, a lymph node biopsy was also performed, which revealed a T-cell lymphoblastic leukemia/lymphoma, BCR/ABL1 positive, with clonal evolution. This is a very rare manifestation of CML in blast crisis with no standard treatment and with poor outcomes despite chemotherapy or allogeneic stem cell transplant. Given its rarity, it would be difficult to develop standard chemotherapy protocols. We believe the treatment for this condition should be similar to any lymphoid blast crisis. The patient was treated with induction chemotherapy (hyper-CVAD regimen) plus dasatinib for 3 cycles followed by sibling-donor allogeneic stem cell transplant and is currently on maintenance dasatinib and has minimal residual disease at this time.

## 1. Introduction

Chronic Myelogenous Leukemia (CML) usually presents in chronic phase, followed by an accelerated phase and a blast phase, if untreated. The blast phase can manifest as myeloid (in about 70% of cases) or lymphoid blast crisis, with the B-cell lineage being more common. CML in blast crisis of T-cell lineage is a very rare manifestation, with only a handful of cases reported. There is no standard treatment for this entity and these patients, unfortunately, have poor outcomes. We report a unique case of CML blast crisis with T-cell lymphoid lineage in a middle-aged male and discuss this entity.

## 2. Case

A 49-year-old man presented to our hospital with a history of night sweats, left-sided abdominal pain, weight loss, and recurrent episodes of streptococcal pharyngitis. He reported first noticing the left-sided abdominal pain about 8 months prior to presenting to our hospital, but it worsened over the past two months.

On examination, the patient was found to have cervical, supraclavicular, and inguinal lymphadenopathy as well as splenomegaly. He had a white blood cell count of 200,400/*μ*L, with a left shift and 3% blasts. The hemoglobin, platelet count, and chemistries were all within normal limits. The imaging revealed multiple enlarged lymph nodes in the neck, mesentery, retroperitoneum, and inguinal as well as a massive splenomegaly (28 cm).

**Figure 1:**
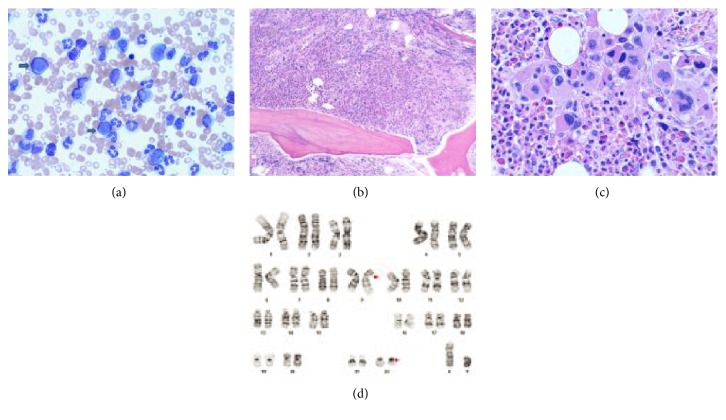
Peripheral blood leukocytosis with left shift and blasts (arrows) (a) Wright stain, ×400. Hypercellular bone marrow with myeloid and megakaryocytic hyperplasia; blasts < 5% (b) H&E, ×100. Cluster of atypical megakaryocytes in bone marrow (c) H&E, ×400. Abnormal bone marrow karyotype: 46,XY,t(9;22)(q34;q11.2)[19]/47,idem,+8 [1] (d).

**Figure 2:**
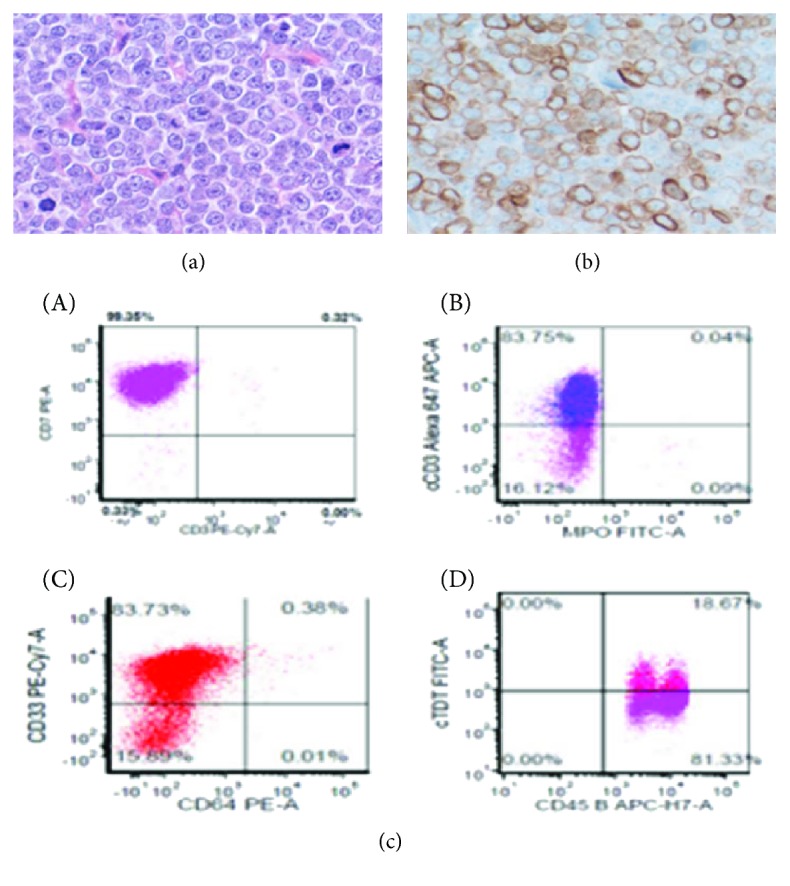
Cervical lymph node with diffuse effacement by blasts (a) H&E, ×400. Immunohistochemistry, anti-CD3 (b) ×400. Flow cytometry: neoplastic cells are positive for CD7 and negative for surface CD3 (c-A), positive for cytoplasmic CD3 and negative for MPO (c-B), positive for CD33 (c-C), and partially positive for TdT (c-D).

**Figure 3:**
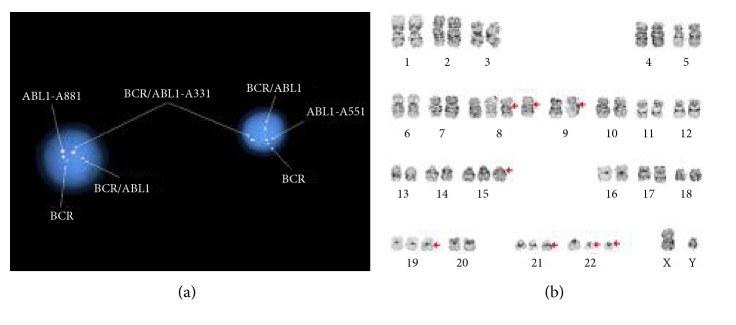
Cervical lymph node: BCR/ABL1 detected by FISH (a). Abnormal lymph node karyotype: 51-53,XY,+8,+8,t(9;22)(34;q11.2),+15,+19,+21,+21,+der(22)t(9;22)[cp20] (b).

Peripheral blood analysis for BCR-ABL1 by PCR revealed the presence of p210 (b3a2) transcript at 24.991%. The bone marrow biopsy showed hypercellular marrow with striking granulocytic and megakaryocytic hyperplasia with atypical megakaryocytes and moderate reticulin fibrosis (Figure 1(a)–1(c)). Left-shifted myeloid maturation with 1.5% blasts was detected by flow cytometry. Fluorescent in situ hybridization (FISH) analysis and karyotyping (Figure 1(d)) confirmed presence of BCR-ABL1 translocation. We started the patient on hydroxyurea for cytoreduction, with a plan to initiate a tyrosine kinase inhibitor (TKI) therapy. However, given the generalized lymphadenopathy, we were concerned about extramedullary (lymph node) blast crisis and included a cervical lymph node biopsy in his work up. This showed diffuse nodal effacement by blasts that were positive for CD3 and negative for CD20, CD34, and myeloperoxidase by immunostaining (Figures 2(a) and 2(b)). The flow cytometry analysis detected an abnormal T-cell phenotype expressing CD45, cCD3, bright surface CD7, CD43, partial CD2, two myeloid markers (CD13 and CD33), dim/partial TdT, and, to a lesser extent, CD1a, while lacking sCD3, CD4, CD8, CD5, and defining markers for myeloid, monocytic, and B-lymphoid lineage (MPO-, CD14-, CD64-, CD11b-, CD19-, and CD20-) (Figure 2(c)). The blasts were positive for BCR-ABL1 by FISH and karyotyping (Figures 3(a) and 3(b)), with additional chromosomal abnormalities indicating clonal evolution of CML with T-cell blast crisis. T-cell gene rearrangement on the lymph node was negative; however, that did not rule out T-cell blast crisis.

The patient was transferred to a higher-care facility with initiation of dasatinib and hyper-CVAD chemotherapy regimen. He completed 3 cycles of hyper-CVAD and underwent sibling-donor stem cell transplant and is on maintenance dasatinib. He is 5 months from diagnosis and is in hematological and cytogenetic remission but has minimal residual disease.

## 3. Discussion

Chronic Myelogenous Leukemia (CML) is a myeloproliferative hematopoietic neoplasm, characterized by a balanced translocation of Abelson gene on chromosome 9q34 with breakpoint cluster region gene on chromosome 22q11.2 (BCR-ABL1) [1]. About 85–90% of new CML patients are diagnosed in the chronic phase, while blast crisis occurs in about 10% of patients [1]. In about 20–30% of CML blast phase, the blasts are lymphoid and usually of B-lineage. CML blast phase with T-cell lymphoid lineage is extremely rare with about 50 case reports of this particular entity being reported with the largest case series by Raanani et al. [2]. The rate of blast transformation of CML is about 1.5% per year, which is significantly better compared to the preimatinib era [3]. Median survival of patients in blast crisis is poor. Jain et al. reported approximately 500 cases of CML blast crisis with only 0.01% being of T-cell lymphoid lineage, and the survival varied from 4 months to 49 months [4].

It is proposed that blast transformation of CML is triggered by molecular or genetic mutations, such as trisomy 8, trisomy 19, isochromosome 17, t(3;21), mutations in p53, RB gene, RAS pathway, or p16/ARF pathway mutations [5]. CML blast crisis of T-cell lineage can be difficult to differentiate from de novo BCR-ABL1-positive T-cell Acute Lymphoblastic Leukemia (T-ALL) and BCR-ABL1-positive bilineage leukemia [6, 7]. Some features of de novo BCR-ABL1-positive T-cell ALL are bone marrow involvement, minor BCR breakpoint mutations, TCR gene rearrangement mutations, children or adolescent age group, and no prior history of CML [2]. In early immature T-cell neoplasms (like T-ALL), a clonal T-cell gene rearrangement (TCR) is not always present, and hence, its absence does not rule out a T-cell blast crisis [8]. BCR-ABL1 positivity in both myeloid and lymphoid cells, rather than just in the lymphoid component, as it was detected in our case, would support the diagnosis of CML in T-cell blast crisis [9].

The reported clinical features in cases of T-cell blast crisis include B symptoms and lymphadenopathy; however, many patients are diagnosed due to abnormal blood work results. It is important to biopsy both the bone marrow and lymph nodes, as the lymph nodes may be the initial site of blast transformation. The timing of developing T-cell blast crisis from CML chronic phase is unclear. In a case series reported by Aguayo et al., the time ranged from 3 months to 48 months [10]. However, conversion to blast crisis happening earlier than 3 months has been reported [11]. There have been also reports of T-cell blast crisis occurring within 1 month of stopping imatinib therapy, which makes the pathophysiology of sudden blast crisis still relatively unknown [12]. Our patient was diagnosed with extramedullary (nodal) T-cell lymphoblastic transformation of CML at presentation.

The treatment of this entity includes approved acute lymphoblastic leukemia chemotherapy regimens with or without tyrosine kinase inhibitors such as imatinib. Complete molecular and cytogenetic response with this regimen has been reported [13, 14]. Allogeneic stem cell transplantation has had success in long-term remission in one reported case [15]. Most patients, unfortunately, relapse early after induction chemotherapy or allogeneic stem cell transplant, making survival rates dismal for this disease. We would recommend induction therapy with acute lymphoblastic leukemia chemotherapy regimens with a TKI. All patients should be considered for allogeneic stem cell transplant and maintenance TKI. Our case is a unique presentation of CML blast crisis, which unfortunately has an aggressive biology.
